# Data Resource Profile: The Scottish National Prescribing Information System (PIS)

**DOI:** 10.1093/ije/dyw060

**Published:** 2016-05-10

**Authors:** Samantha Alvarez-Madrazo, Stuart McTaggart, Clifford Nangle, Elizabeth Nicholson, Marion Bennie

**Affiliations:** ^1^ Strathclyde Institute of Pharmacy and Biomedical Sciences, University of Strathclyde, Glasgow, UK and; ^2^ Public Health and Intelligence Strategic Business Unit, NHS National Services Scotland, Edinburgh, UK

## Data resource basics

The Prescribing Information System (PIS) provides an infrastructure for pharmacoepidemiology and pharmacovigilance research, attracting growing attention due to its unique combination of characteristics. Compared with several databases worldwide offering national population coverage and record linkage, ^1–3^ PIS covers all National Health Service (NHS) prescriptions prescribed, dispensed and reimbursed within the community setting, covering in Scotland a total population of 5.3 million residents.

PIS provides summary information on reimbursed medicines from 1993, and it also gives access to individual prescribing and dispensing data since 2009 with the incorporation in the system of a unique number, specific to NHS Scotland, called the Community Health Index (CHI) number (the PIS evolution process is shown in Supplementary Figure 1 , available as Supplementary data at *IJE* online). Moreover, the presence of this number allows the linkage of PIS data to other health records data, thus describing a patient’s pathway through the healthcare system. This dataset addresses the demand for longitudinal data at a population level, captured through routine clinical systems required to understand the chronology of drug use and health outcomes, complementing the evidence generated through clinical trials and drug surveillance schemes. ^4–6^

### Data resources and population coverage

**Figure 1.**
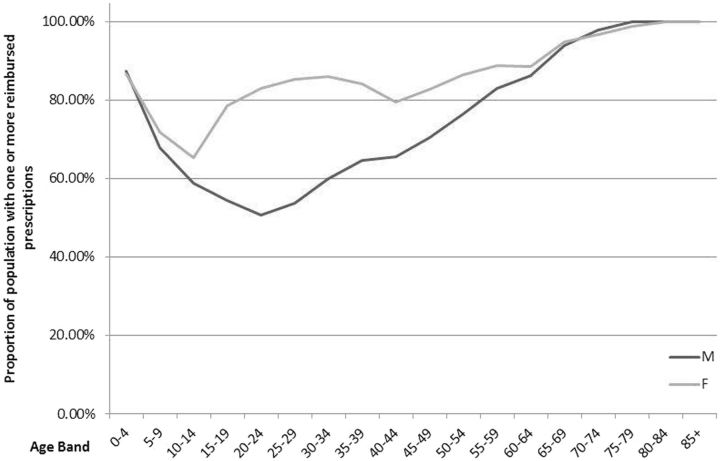
Demographic characteristics of patients in PIS with a reimbursed prescription in 2014.

PIS holds information for over 1.6 billion prescriptions reimbursed in the community from January 1993 to 2014, over 507 million items prescribed and over 344 million items dispensed from 2009 to 2014. ^7^ The CHI capture rate, which shows the availability of patient-level data, is almost 100% for the prescribed and dispensed items. Only those with an invalid CHI are not included. For the reimbursed items, the rate has increased from 87.7% in 2009 up to 95.6% in 2014. In addition to items per patient, PIS also covers information on active NHS prescribers and approximately 1200 community pharmacies. In the calendar year of 2014, more than 70% of all men and 85% of all women had one or more prescriptions reimbursed. The proportion is similar in children (0–4 years) and people > 65 years old, regardless of gender. However, there is a higher proportion of adult women in the population with reimbursed prescriptions than men ( Figure 1 ).

## Data collected

### Funding sources

PIS is an NHS data system and is funded from the public monies available to the NHS. It is hosted within a national information warehouse managed by NHS National Services Scotland (NHS NSS). Current work to develop an improved PIS research-ready analysis platform is supported by the Farr Institute @ Scotland and its 10-funder consortium. Research studies using PIS, as well as other datasets, are funded by a variety of sources including the Farr Institute, Chief Scientist Office, Medical Research Councils and UK charities.

### Dataset production

**Figure 2.**
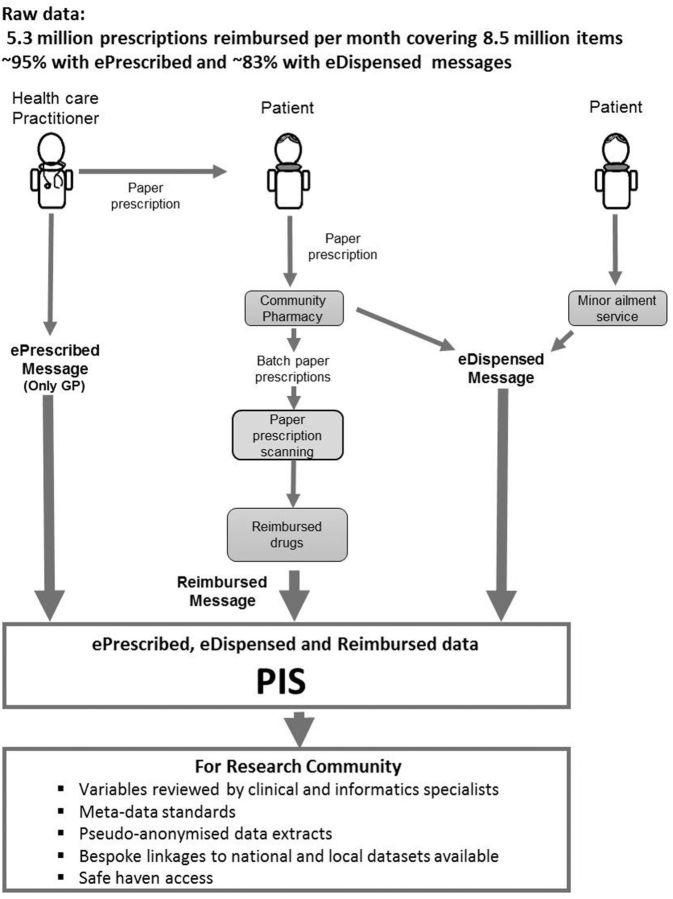
PIS prescribing, dispensing and reimbursed data from raw (2014) to researcher – ready access.

Figure 2 illustrates the sustainable journey of collecting, prescribing and dispensing raw data at a population level from three data sources—ePrescribed, eDispensed and Reimbursed messages—which form the basis of PIS, and the data available for the researcher community.

Prescriptions are written by a range of healthcare practitioners including general practitioners (GPs), nurses, dentists, pharmacists and an expanding range of other non-medical prescribers. In all cases, a paper form is provided to the patient that they may take to the pharmacy of their choice. In the case of GP prescribing, an electronic prescription message (ePrescribed) is also generated and, where this is the case, the dispensing pharmacist has the option to also submit an electronic dispensed message (eDispensed) to support the claim for reimbursement. Once the prescription has been dispensed by the pharmacy and collected by the patient, the paper forms are submitted in monthly batches to NHS NSS for reimbursement. Information from the paper form is captured by high-speed scanners using optical character recognition, supplemented by human operators. Where electronic messages also exist for the prescription, then these act as the primary source of information in preference to that captured by scanning. The output from this process forms the reimbursed message. As GPs account for more than 95% of prescribing in primary care, the great majority of prescriptions processed have at least one accompanying electronic source. Data extracts of PIS can be made available for researchers upon request, subject to relevant governance approval.

### Frequency of data collection

Data generation takes place as part of routine prescribing by health professionals and dispensing in the community pharmacies in Scotland. The frequency of data generation is determined by the needs and conditions of the patient, but long-term repeat medicines are typically prescribed and dispensed at 28- or 56-day intervals. Reimbursement data are processed and loaded on a monthly cycle and are available approximately 2 months after the prescription was dispensed and collected by the patient. The ePrescribed and eDispensed data are loaded daily and are available 24 h and 14 days after the prescription was written and dispensed, respectively.

### Measures

Data variables included in PIS fall into four main categories: (i) patient-specific data; (ii) prescriber data; (iii) dispenser data; and (iv) drug data. More detailed information about the PIS fields for researchers is available from the Information Services Division Scotland website. ^8^

#### 

##### Patient-specific data

Patients included in the database are identified by their community health index (CHI) number. As well as age and gender, CHI enables the prescription record to be populated with data from the CHI national register when requested. This register defines the patient’s place of residence and indicates whether or not the patient is in a care home. Geography-related information, such as the Scottish Index of Multiple Deprivation (SIMD) ranking and urban/rural classification, is also available.

##### Prescriber data

Prescribers have a unique identifier that identifies the profession and the location where the prescription was written. For GP practices, the number and demographics of the registered patients is also available.

##### Dispenser data

Each dispensing organization is assigned a dispenser type, e.g. community pharmacy, and geographical location.

##### Drug data

For each reimbursed prescription, individual variables describing the approved name (normally International Nonproprietary Names (INN)), product name, formulation and strength are available. Quantity information is available as number of tablets, etc., supplied and also expressed in defined daily doses (DDDs). Information is also available about instalment dispensing and the number of instalments, including how many were supervised (e.g. opiate substitution therapy). The database captures information from the time the drug is prescribed to the time (whether or not) it has been dispensed or reimbursed. Each drug product is also identifiable by an extended *BNF* ( *British National Formulary* ) code. ^9^ This is a structured code that classifies medicines according to therapeutic use as described in the *BNF* , ^10^ and the extended code differentiates between proprietary and generic products and formulation and strength.

Drug data within ePrescribed and eDispensed messages are expressed using codes and descriptions from the NHS dictionary of medicines and devices (dm+d), which is a UK extension of SNOMED-CT. ^11^ In addition, electronic messages include the prescribed dose instructions, as free text, and so these are also available for reimbursed prescriptions for which an electronic message exists.

### Data linkage

The CHI number is a unique numeric identifier, allocated to each patient on first registration with the Scottish healthcare datasets, but not social datasets. It is a 10-character code consisting of the 6-digit date of birth (DDMMYY), two digits, a 9th digit which is always even for females and odd for males and an arithmetical check digit. ^12^ The inclusion of this unique identifier in PIS allows for accurate health data linkage at an individual level with well-coded national and local databases, enabling studies to be conducted across the entire lifespan of individuals and populations. Further detail on the national databases catalogue, including hospital records (SMR00 and SMR01), maternal and neonatal records (SMR02), the Scottish Cancer Registry (SMR06) and mortality records, is available at [ www.ndc.scot.nhs.uk ]. Subject to approval by the Public Benefit and Privacy Panel for Health and Social Care (PBPP), other external datasets, e.g. clinical trials, local health board data, disease registries and non-health administrative data, can also be linked.

To comply with information governance procedures and preserve patients’ confidentiality, the OECD Guidelines on Human Biobanks and Genetic Research Databases ^13^ are followed and the electronic Data Research and Innovation Service (eDRIS) team provides pseudo-anonymized data extracts for researchers’ use. CHI numbers are replaced by unique study numbers and personal identifying information is removed. This is achieved in a secure manner by having different teams handling patient identifiers and study variables, and then performing the linkage. ^14^

Having national datasets with an outstanding quality of recording events such as hospitalizations, surgical procedures and underlying causes of mortality, record linkage has successfully been carried out in Scotland in areas such as diabetes. ^15^ This goes along the same lines of successful record-linkage programmes in the UK, done with other data resources focusing on cardiovascular diseases, primary care and a subset of dispensed or prescribed prescriptions in the population. ^16^^,^^17^

## Data resource use

PIS has the flexibility to be used as a stand-alone dataset or connected to further datasets by using electronic record linkage. Cohorts can either be identified according to the type of drug/drug group of interest or patient groups can be selected according to disease or other characteristics, and using record linkage their prescriptions in primary care can be studied. The possibility of creating groups is not limited to those receiving a prescription or in PIS as, in addition, matched (or unmatched) control groups—e.g. from the CHI registry or other external datasets—can also be created. Table 1 provides examples of studies conducted using PIS, ranging from drug use to evaluation of drug effects, guidance about drug prescribing and health economics. ^18–37^ By example, the knowledge generated from PIS has already generated a change in the Scottish national antibiotic policy recommendation for orthopaedic surgical prophylaxis and has been used to evaluate interventions to improve high-risk prescribing in primary care. ^19^^,^^20^^,^^29^

**Table 1.** Examples of projects by study type and extent of record linkage using PIS, 2009–15

Project title	Drug utilization study	Effect study	Health economics	Other	Linkage to other databases	Published (P)/Ongoing (O)
Trends of utilization or enhancement to prescribe proton pump inhibitors, statins, angiotensin receptor blockers and antipsychotics ^21–23^	✓					**P**
Factors influencing prescribing of antidepressants ^33^	✓					**P**
Burden of irritable bowel syndrome ^31^	✓					**P**
Primary care prescribing indicators ^28^				✓		**P**
Polypharmacy Guidance ^32^				✓		**P**
Prescribing of oral generic risperidone – findings across Europe ^26^	✓			✓		**P**
Policies to enhance prescribing efficiency in Europe ^27^				✓		**P**
Socioeconomic deprivation and primary care antibiotic prescribing ^25^	✓					**P**
Reduction in broad-spectrum Gram-negative agents ^30^	✓			✓		**P**
Risk of acute kidney injury with gentamicin as surgical prophylaxis ^20^		✓				**P**
Feedback to Improve Primary Care Prescribing Safety (EFIPPS Study) ^19^^,^^29^	✓			✓		**P**
Opioid prescribing ^35^	✓					**O**
Methadone prescribing in Scotland ^24^	✓					**O**
Use of national, linked administrative health data to quantify the association between antimicrobial prescription and *Clostridium difficile* infection: a population based matched case control study (Unpublished)	✓	✓			✓	**O**
Impact on prescriptions and hospital admission of prescription fee abolition in Scotland ^36^				✓	✓	**O**
The Greater Glasgow and Clyde Biologics Cohort ^18^	✓	✓			✓	**O**
Utilization, clinical effectiveness and safety of oral anticoagulants and oral antiplatelets in Scotland ^34^^,^^37^	✓	✓	✓		✓	**O**

## Current developments

The FARR Institute @ Scotland, an NHS/academic collaboration, is enabling new datasets for research and improved patient care including PIS, alongside a new national GP primary care dataset (SPIRE), imaging (PACS Images) and laboratory dataset (SCI-Store). ^38^^,^^39^ The investment in PIS is supporting improved drug coding and connection to Anatomical Chemical Classification (ATC) coding to support cross-national studies, as well as transformation of drug dosage instructions into structured fields to support the interpretation and quantification of drug exposure and measurement of patient adherence. A prototype algorithm has been developed and tested in response to the demand for drug dosage metadata. ^24^

Further development is underway to apply the algorithm to all prescription instructions held within PIS, thereby enabling direct querying of data by drug dose, unit and frequency of expected consumption. This work has supported a preferred partner status for future rapid pharmacovigilance studies in collaboration with the European Medicines Agency.

## Strengths and weaknesses

### Strengths

#### Coverage

The NHS in Scotland is a publicly-funded healthcare system and is universally used by the 5.3 million people living in Scotland. The dataset can be censored for death and patients who have de-registered with the NHS in Scotland, through linkage to other datasets. The NHS in Scotland provides universal coverage; thus PIS is representative of all age, sex and socioeconomic groups and geographies, is free from any selection bias and allows for the detection of rare events.

By the end of 2014, 95% of records captured included the unique person identifier, and so data from PIS can be accurately linked to a wide range of other health and non-health datasets.

PIS is one of the few nationwide databases which include routinely collected data on prescribed items and whether they were dispensed and reimbursed or not. This offers the possibility of studying treatment regimens and their outcomes in the population, polypharmacy and primary non-adherence although, in common with most other prescription registries, there is no information about when or whether the patient actually consumed the dispensed medicine.

Another feature of its coverage is the availability of drug dosage information, which is necessary for studies with dose-dependent association between exposure and effect.

#### Completeness

Prescriptions must be submitted for payment in order that the dispenser can be reimbursed for the products supplied, providing a strong incentive to do so. Data therefore have a high level of completeness. However, although the availability of individual level data is high, this is influenced by the type of health care practitioner prescribing and/or service delivery models: in 2014, CHI capture by healthcare practitioner varied from 98.7% for GP prescribers to 1.6% for dentists; and for the same year, the CHI capture rate of immunological products and vaccines was 71.5% compared with 98.6% for cardiovascular medicines. Further data processing by researchers may be required according to the completeness for specific studies.

#### Data quality

The capture by PIS of electronic prescription and dispensing messages has resulted in an improvement in data quality by removing the majority of manual data entry processes. In addition, the millions of raw records go through more than 10 stages of quality checking before and after they are submitted to PIS. ^40^

#### Follow up

Longitudinal studies are feasible, both for cohorts and for follow-up to clinical trials, thus enabling research about use of medicines and healthcare resources in chronic conditions as well as monitoring long-term outcomes. Considering 2009 as the year in which CHI capture increased, the present median follow-up is 6–7 years per patient.

### Weaknesses

#### Data not captured

PIS does not capture information about retail sales of over-the-counter (OTC) medicines. It does however include supplies of OTC medicines made to eligible individuals by community pharmacists under the Minor Ailments Service (MAS). ^41^ Medicines administered during inpatient hospital stays and upon discharge for short-term use, and some specialist drugs for chronic use such as biologicals and growth hormone therapy are not captured, nor are outpatient supplies made by the hospital service. Information about vaccines is variable: some of this is captured through an alternative childhood immunization database. ^42^

There is no information about diagnosis or indication for treatment included on prescriptions, and so this information is not available in PIS. Data from primary care (SPIRE) may become available for linkage. ^39^ In many cases this limitation can be overcome by linkage to secondary care or local datasets, and case identification can be optimized with the corresponding algorithm as has been done in other data resources. ^43^^,^^44^

#### Data availability

More than 87% capture of the patient identifier was achieved in 2009, but this figure falls away rapidly from 68% in 2008 to less than 1% in 2003. Therefore longitudinal studies with PIS individual-level data are recommended from 2009 onwards. PIS currently is suitable for most research purposes; however, the processing lag and variation in the frequency of data loads means that it is unable to support real-time medication reconciliation studies.

## Data resource access

Researchers can access non-identifiable (but linkable) data from PIS by contacting the eDRIS service run by NHS NSS [ www.isdscotland.org/Products-and-Services/eDRIS/ ]. Each research proposal is assigned an experienced research coordinator who will guide the research team through the selection of appropriate data items and any limitations applicable to the proposed study, as well as guidance on completion of the PBPP application process for approval to link data. Once the requested data are approved by the PBPP, data are extracted and provided to researchers through personal accounts in national or local research portals (safe havens) with secure access and storage. ^45^^,^^46^ Currently PIS data are available to researchers based in recognized UK institutions. The PBPP can consider on a case-by-case basis to provide data outside the UK. For more information regarding application and costs, contact the eDRIS team at [NSS.eDRIS@nhs.net].

## Funding

This study was supported by the Farr Institute (grant no. MR/K007017/1). The Farr Institute @ Scotland is supported by a 10-funder consortium: Arthritis Research UK, the British Heart Foundation, Cancer Research UK, the Economic and Social Research Council, the Engineering and Physical Sciences Research Council, the Medical Research Council, the National Institute of Health Research, the National Institute for Social Care and Health Research (Welsh Assembly Government), the Chief Scientist Office (Scottish Government Health Directorates) and the Wellcome Trust.

Profile in a Nutshell

- In 2009, the Prescribing Information System (PIS) was developed as a national individual-level dataset of prescriptions prescribed, dispensed and reimbursed within the community setting in Scotland, covering the 5.3 million residents.
- The dataset provides information about patient, prescriber and dispenser characteristics, and the drugs prescribed, dispensed and reimbursed (> 98% of the items prescribed by general practitioners have individual-level records).
- The availability of aggregated and patient-level data, linkable to a variety of local and national databases, supports retrospective and prospective pharmacoepidemiological studies at different levels of complexity.
- Evolving PIS developments (including enhanced drug coding and interpretable dose instructions) will provide better intelligence on the safety and effectiveness of medicines in routine clinical practice.
- Data are available upon request to the electronic Data Research and Innovation Service [NSS.eDRIS@nhs.net].

## Supplementary Material

Supplementary Data
