# Supplementary material for: Data Resource Profile: The Scottish National Prescribing Information System (PIS)
Source: Int J Epidemiol. 2016 May 10;45(3):714–715f. doi: 10.1093/ije/dyw060 (PMC5005947; doi:10.1093/ije/dyw060)
Supplement: Supplementary Data [file dyw060_supplementary_data.zip › ije-2015-12-1653-File006.docx]

**Supplement**

Supplementary Figure 1 illustrates the historical development of PIS from a cost and volume dataset, supporting routine drug utilisation studies, to the present evolving capacity to undertake individual level pharmacoepidemiology studies.

**Supplementary Figure 1. Evolution of the Prescribing Information System.**

**
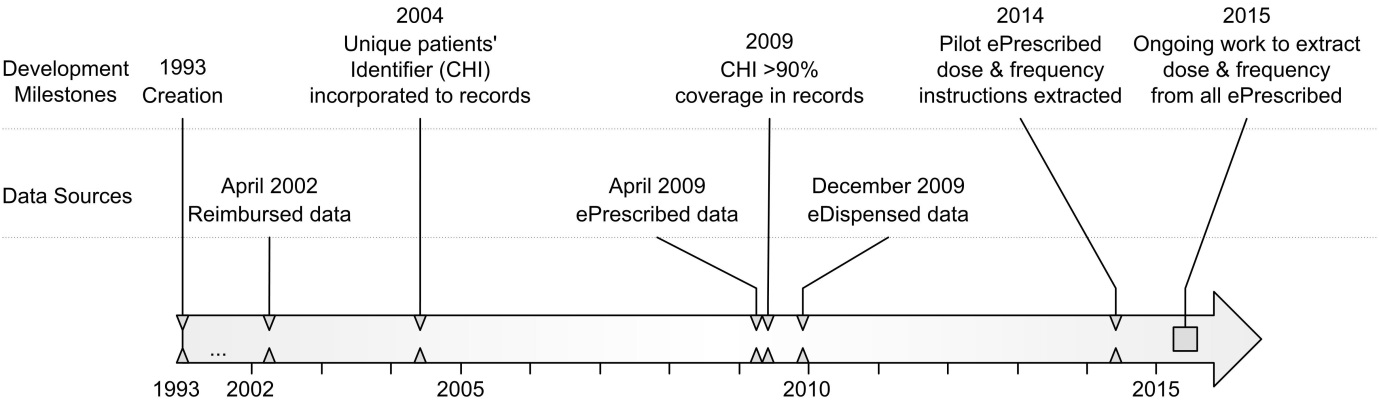
**
